# Supplementary material for: SH3-domain mutations selectively disrupt Csk homodimerization or PTPN22 binding
Source: Sci Rep. 2022 Apr 7;12:5875. doi: 10.1038/s41598-022-09589-9 (PMC8989918; doi:10.1038/s41598-022-09589-9)
Supplement: Supplementary file 1 — Supplementary Figures. [file 41598_2022_9589_MOESM1_ESM.docx]

**Supplementary Materials**

SH3-domain mutations selectively disrupt Csk homodimerization or PTPN22 binding

Ben F. Brian IV, Frances V. Sjaastad, and Tanya S. Freedman

**
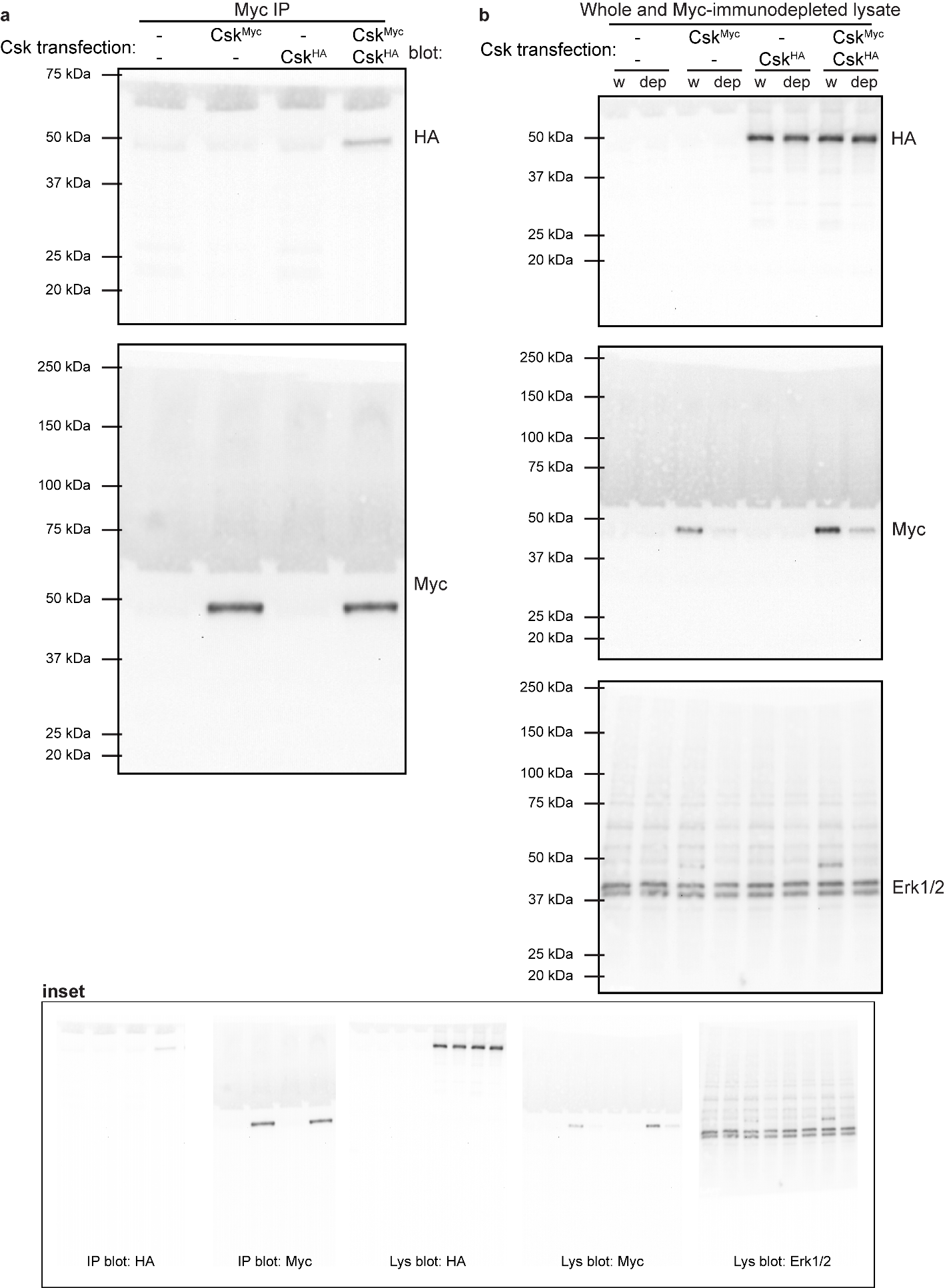
**

**Supplementary Fig. S1. Uncropped images from Fig. 1. (a)** Immunoprecipitate blots from Fig. 1a. **(b)** Lysate blots from Fig. 1b. **inset:** Different exposures of gels above. **inset:** alternative image exposure.

**
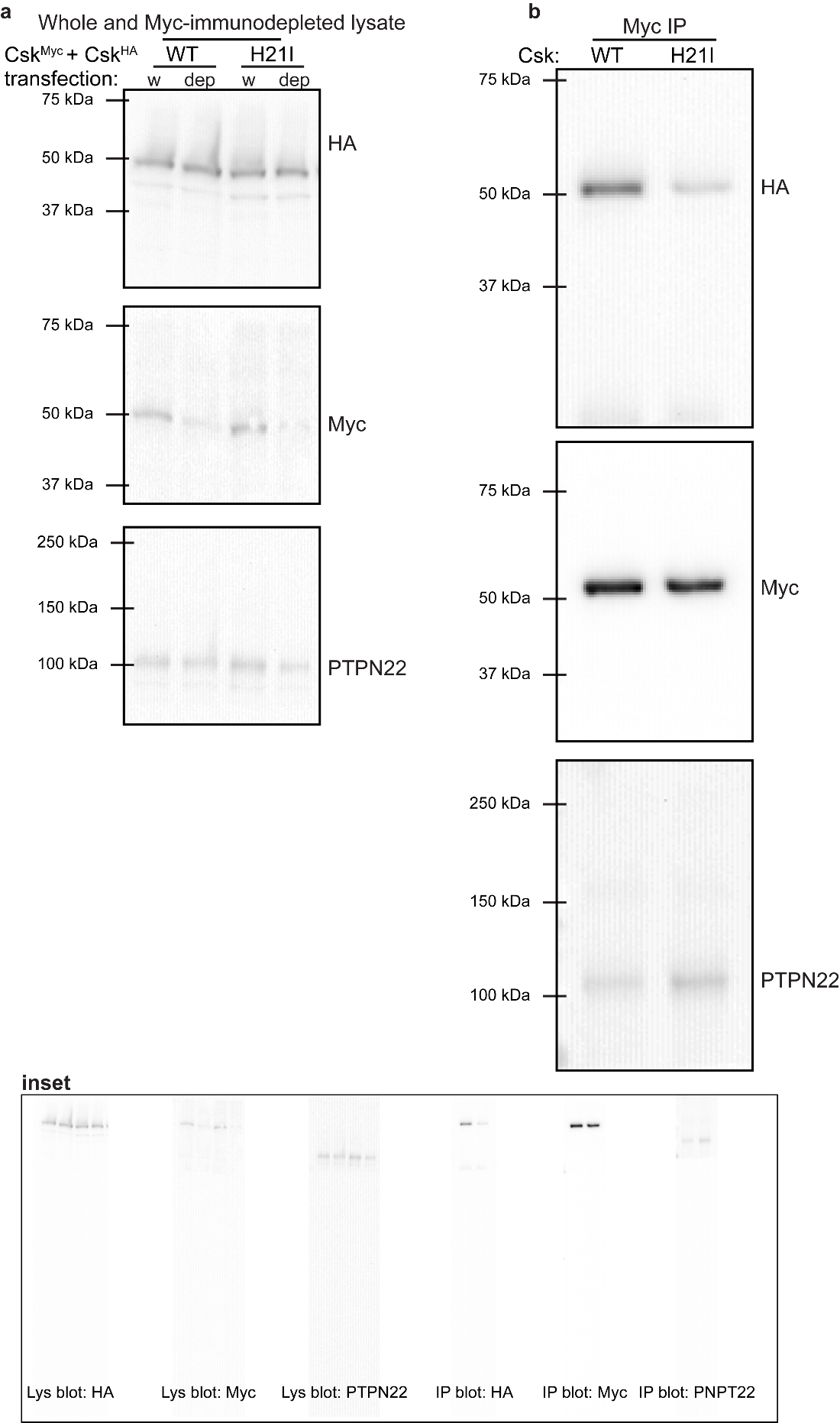
**

**Supplementary Fig S2. Uncropped images from Fig. 2. (a)** Lysate blots from Fig. 2c. **(b)** Immunoprecipitate blots from Fig. 2d. **inset:** alternative image exposure.

**
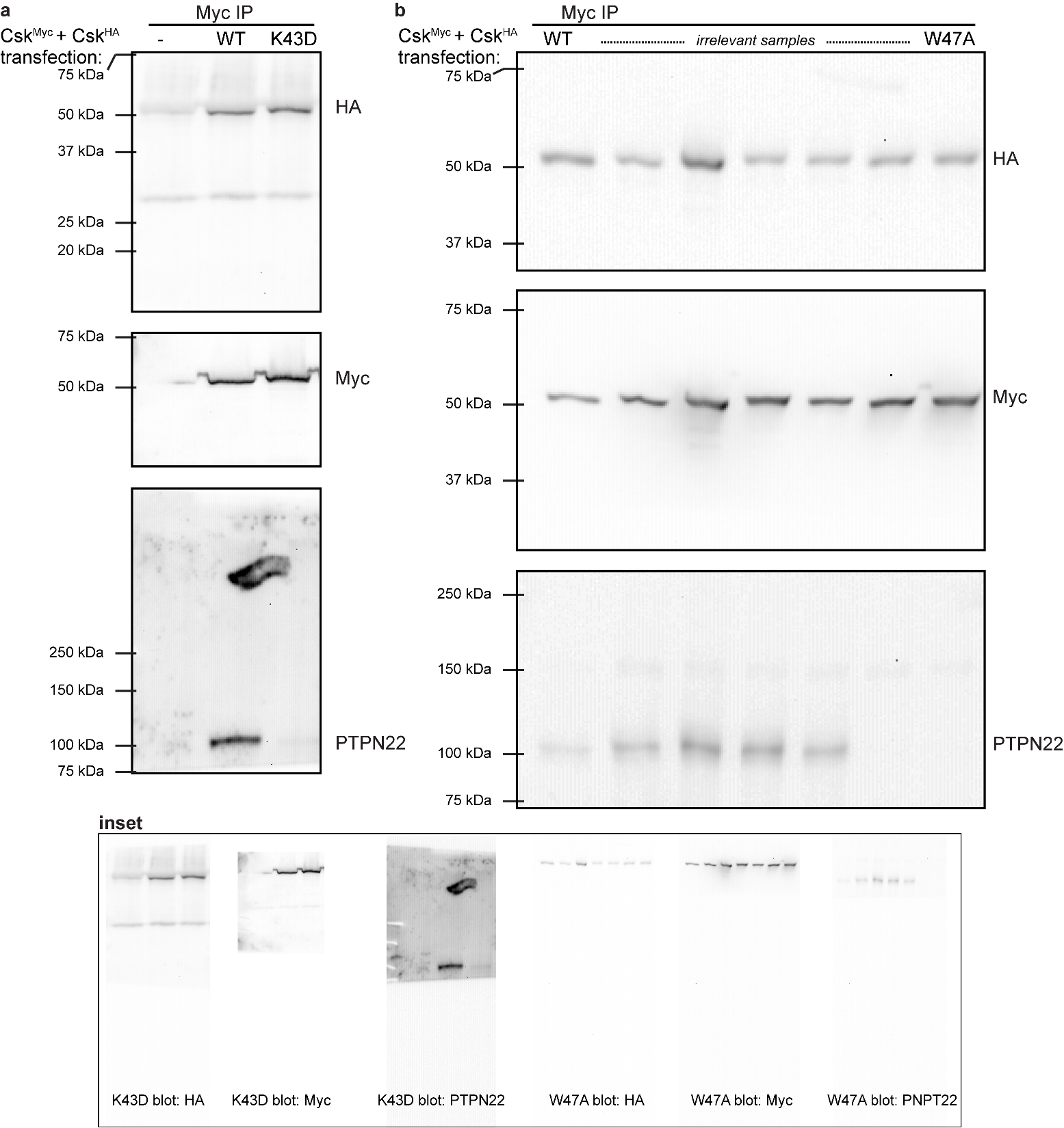
**

**Supplementary Fig S3. Uncropped images from Fig. 4. (a)** K43D immunoprecipitate blots from Fig. 4b. **(b)** W47A immunoprecipitate blots from Fig. 4d. **inset:** alternative image exposure.
